# Unlocking the Anti-Inflammatory Potential of Sourdough: Phytochemical Profile, Functional Investigation, and Molecular Docking Insights into Key Bioactive Compounds

**DOI:** 10.1007/s11130-025-01345-4

**Published:** 2025-04-08

**Authors:** Asma Cherbal, Yousra Abboud, Rayane Lakroun, Dina Nafa, Erdi Can Aytar, Salima Khaldi

**Affiliations:** 1 Department of Molecular and Cell Biology, Faculty of Nature and Life Sciences, University Dina of Jijel, Jijel, 18000 Algeria; 2 Biomathematics, Biophysics, Biochemistry, Scientometry Laboratory, Faculty of Nature and Life Sciences, University Abderrahmane Mira-Béjaïa, Béjaïa, 06000 Algeria; 3 Department of Horticulture, Faculty of Agriculture, Usak University, Uşak, 64200 Turkey

**Keywords:** Antioxidant activity, Anti-inflammatory activity, GC-MS analysis, Molecular docking, Sourdough

## Abstract

Sourdough, a fermented food product, has garnered increasing interest for its potential health benefits. This study comprehensively evaluated the anti-inflammatory and antioxidant potential of sourdough extract in vitro. Phytochemical analysis revealed a significant presence of phenolic compounds (0.276 ± 0.0196 mg GAE/g), and GC/MS analysis identified key bioactive compounds, including glycerin, lactic acid, thymol, phthalic acid, linoleic acid, and caprylic acid. The sourdough extract demonstrated potent anti-inflammatory activity, evidenced by its ability to inhibit protein denaturation (92.27 ± 0.66%), protease activity (26.70 ± 1.36%), and red blood cell hemolysis (22.93 ± 0.73%), suggesting a protective effect against cellular damage and inflammation. Furthermore, the extract exhibited considerable antioxidant capacity, as demonstrated by DPPH (14.29 ± 0.04%), H2O2 (81.61 ± 2.98%), and FRAP (96.26 ± 0.03%) assays. Molecular docking studies provided mechanistic insights into the anti-inflammatory effects, revealing that thymol and phthalic acid, identified in the extract, exhibited strong binding affinities with the IL-1β signaling complex (PDB ID: 4DEP), with binding energies of -6.6 kcal/mol and − 5.5 kcal/mol, respectively. These findings suggest that these compounds may contribute to the modulation of inflammatory pathways. This research highlights the significant anti-inflammatory and antioxidant potential of sourdough, suggesting its potential as a functional food for promoting human health. Moreover, it underscores the contribution of specific bioactive compounds to the observed effects, paving the way for further investigation into the therapeutic applications of sourdough.

**Supplementary Information:**

The online version contains supplementary material available at 10.1007/s11130-025-01345-4.

## Clinical Trial Number

Not applicable.

## Introduction

Inflammatory diseases are a leading cause of global mortality and morbidity, with diet recognized as a key environmental factor influencing their onset and progression [1]. While current anti-inflammatory medications, such as nonsteroidal anti-inflammatory drugs (NSAIDs) and steroidal anti-inflammatory drugs, demonstrate efficacy, their use is often limited by severe adverse effects and high costs. For instance, certain NSAIDs have been withdrawn from the market due to their association with fatal cardiovascular and hepatic toxicity [2]. These limitations have spurred interest in identifying natural, cost-effective alternatives with fewer side effects.

Fermented foods, particularly those derived from cereals, have garnered significant attention for their potential health benefits. Among these, sourdough fermentation has been shown to enhance the bioavailability of bioactive compounds, including polyphenols, peptides, and organic acids, which exhibit antioxidant, antihypertensive, and anti-inflammatory properties [3, 4]. Despite these promising findings, the specific effects of sourdough fermentation on the functional properties of wheat and its potential mechanisms of action remain underexplored [5].

Probiotics have emerged as key players in the health benefits associated with fermented foods. These live microorganisms, when consumed in adequate amounts, confer health benefits by modulating gut microbiota, enhancing gut barrier function, and producing bioactive metabolites with anti-inflammatory and antioxidant properties. For instance, *Lactiplantibacillus plantarum* K25 has demonstrated significant cholesterol-lowering and angiotensin I-converting enzyme (ACE) inhibitory activities, highlighting its potential in managing cardiovascular and inflammatory conditions [6]. The integration of probiotics into fermented foods, such as sourdough, not only enhances their functional properties but also contributes to the production of bioactive peptides and polyphenols, which synergistically promote health. This underscores the importance of probiotics as a dietary intervention for mitigating inflammation and associated chronic diseases.

Polyphenols, a major class of bioactive compounds produced during fermentation, have been extensively studied for their diverse health-promoting effects. These include antioxidant, anti-inflammatory, hypocholesterolemic, and antihypertensive activities, which are mediated through interactions with gut microbiota and modulation of inflammatory pathways [6]. Recent research highlights the bidirectional relationship between gut health and inflammatory diseases, emphasizing the role of dietary interventions in maintaining gut barrier integrity and reducing systemic inflammation [1, 4].

This study aimed to investigate the phytochemical composition of sourdough extract and evaluate its anti-inflammatory and antioxidant potential using in vitro assays. We employed a multidisciplinary approach, including GC/MS analysis, protein denaturation inhibition assays, protease inhibition assays, red blood cell hemolysis assays, and antioxidant activity assessments (DPPH, H_2_O_2_, and FRAP assays). Additionally, molecular docking simulations were conducted to elucidate the mechanisms by which key bioactive compounds, such as thymol and phthalic acid, interact with inflammatory signaling pathways.

By uncovering the molecular mechanisms underlying sourdough’s bioactivity, this research seeks to contribute to a deeper understanding of its potential as a natural, dietary source of health-promoting compounds.

## Materials and Methods

The material and methods section are presented as Supplementary Material (SM).

## Results and Discussion

The search for natural compounds with anti-inflammatory and antioxidant properties has increased in recent years, owing to their potential therapeutic applications in managing chronic and infectious diseases. Sourdough fermentation, known for its ability to generate bioactive compounds from various natural sources, has emerged as a promising avenue for exploiting these beneficial properties [7]. This study aimed to characterize the phytochemical profile and evaluate the in vitro anti-inflammatory and antioxidant activity of a naturally fermented sourdough extract.

### Phenolic Content of the Sourdough Extract

Total phenolic content was expressed as milligrams of gallic acid equivalents per gram of extract (mg GAE/g). The gallic acid calibration curve (y = 3.7488x + 0.2774, R² = 0.9671) was used for quantification. The extract contained 0.276 ± 0.0196 mg GAE/g. Total flavonoid content was expressed as milligrams of quercetin equivalents per gram of extract (mg QE/g). The quercetin calibration curve (y = 15.251x + 0.333, R² = 0.9894) was used for quantification. The extract contained 0.048 ± 0.0006 mg QE/g. Flavonol content was expressed as milligrams of quercetin equivalents per gram of extract (mg QE/g). The quercetin calibration curve (y = 11.437x + 0.1725, R² = 0.9856) was used for quantification. The extract contained 0.013 ± 0.008 mg QE/g (Table S1).

The phytochemical analysis revealed a significant content of polyphenols, flavonoids, and flavanols in the sourdough extract, with total polyphenols measured at 0.276 ± 0.0196 mg GAE/g of the sourdough extract. This value falls within the range reported by [8]where total polyphenols in various sourdough types ranged from 0.19 ± 0.00 to 0.25 ± 0.06 mg GAE/g. However, other studies have reported higher polyphenol concentrations in sourdough extracts, with [9]finding 1.83 ± 0.02 mg GAE/g. However, it’s crucial to note that the extraction method, sourdough type, and fermentation conditions significantly impact the polyphenol profile, leading to variations across different studies [10].

Several mechanisms contribute to the enrichment of polyphenols during sourdough fermentation, including the hydrolysis of complexed and glycosylated forms by lactic acid bacteria enzymes, the activation of cereal enzymes releasing bound phenolics, and the improved solubilization of phenolic compounds due to system acidification [11].

### Gas Chromatography-Mass Spectrometry Analysis of Sourdough Extract: Composition and Key Components Identified

The sourdough extract exhibited a diverse composition, with 12 distinct compounds identified through qualitative and quantitative analysis (Fig S1Table S2). Glycerin (37.99%) and lactic acid (24.27%) were the most abundant components, followed by di-n-octyl phthalate (20.10%). Other notable components included volatile compounds like thymol (5.65%) and linalol (2.26%), as well as fatty acids like linoleic acid (1.26%) and docosatrienoic acid (3.07%).

Gas Chromatography-Mass Spectrometry (GC-MS) analysis of the sourdough extract identified 12 distinct chemical components, many of which possess beneficial bioactivities including antimicrobial, antioxidant, and anti-inflammatory properties. Thymol significantly contributes to intestinal barrier function improvement and reduces pro-inflammatory cytokine expression. It also exhibits strong antimicrobial efficacy against various pathogens and demonstrates antioxidant properties [12]. Lactic acid, a major organic acid produced by lactobacilli, exerts anti-inflammatory effects by reducing toll-like receptors (TLR)-induced pro-inflammatory responses. Its immunomodulatory action involves increasing IL-1RA production, an anti-inflammatory cytokine that blocks IL-1 receptor signaling and inhibits pro-inflammatory cytokines [13]. Phthalic acid exhibits antioxidant, and larvicidal activities [14]. Glycerin, a simple trihydroxy alcohol, possesses bactericidal and strong virucidal activity [15].

The detection of di-n-octyl phthalate (DOP), comprising 20.10% of the GC-MS profile of the sourdough extract, presents a complex challenge regarding its origin. While DOP is a well-known plasticizer often associated with industrial contamination, the possibility of phthalates occurring naturally in various matrices is an area of active research. Although generally considered anthropogenic contaminants, studies suggest that phthalic acid esters (PAEs), including certain phthalates, can be biosynthesized by various organisms [16]. This raises the crucial point of distinguishing between natural production and contamination, a key consideration in our study. As Thiemann (2021) highlights, differentiating between natural products and contaminants in plant material, where phthalates have been reported, presents a significant challenge due to the ubiquitous nature of these chemicals as environmental pollutants [17].

The complexity of phthalate origins extends beyond plants. Research on algae, for example, reveals that these organisms can both biosynthesize phthalates and bioaccumulate them from the environment [18]. This dual interaction underscores the difficulty in definitively determining the source of phthalates in biological samples. Furthermore, the broader environmental context, including the occurrence and fate of phthalate esters [19], reveals the numerous pathways by which these chemicals can enter various ecosystems, further complicating source identification. Even in seemingly controlled environments, such as indoor spaces, phthalates can originate from diverse sources, including building materials, consumer products, and potentially even plant material [20], highlighting the ubiquitous nature of these compounds.

Critically, in the present study, the sourdough was prepared exclusively in glassware, and no plasticware was used during the preparation or extraction procedures. This significantly mitigates the risk of contamination from these common sources. While we acknowledge that other potential contamination pathways, such as solvents or components of the GC-MS system, cannot be entirely excluded, the absence of plasticware during preparation and extraction strengthens the argument for exploring alternative origins of DOP in our extract.

### The Anti-Inflammatory Activity of the Sourdough Extract

#### Inhibition of Ovalbumin Denaturation

The sourdough extract exhibited a significant protective effect against heat-induced denaturation of ovalbumin, as demonstrated in Fig. S2A. Across a range of concentrations (100 to 1600 µg/ml), the sourdough extract demonstrated a strong inhibitory effect on ovalbumin denaturation. This inhibitory activity increased progressively and significantly with increasing extract concentration. Notably, the sourdough extracts consistently outperformed diclofenac, a known anti-inflammatory drug, at all tested concentrations. At 1600 µg/ml, the sourdough extract achieved an impressive inhibition of 92.27 ± 0.66%, compared to 54.01 ± 0.93% for diclofenac (*p* < 0.0001).

The ability of a substance to inhibit protein denaturation suggests potential anti-inflammatory activity, as tissue damage can be linked to the denaturation of cellular proteins and intercellular substances [21]. The sourdough extract demonstrated significant inhibition of ovalbumin denaturation, reaching a maximum of 92.27 ± 0.66%, surpassing the inhibitory effect of diclofenac (54.01 ± 0.93%). These findings suggest that the sourdough extract possesses potentially superior anti-inflammatory activity compared to diclofenac, a nonsteroidal anti-inflammatory drug known to inhibit biosynthesis of prostanoids, including prostaglandin-E2, prostacyclins, and thromboxanes, key mediators of inflammation [7].

### Stabilization of Red Blood Cell Membrane

The sourdough extract’s protective effect against heat-induced red blood cell hemolysis was investigated and is presented in Fig. S2B. The results demonstrate that the sourdough extract effectively inhibits red blood cell hemolysis at concentrations ranging from 100 to 1600 µg/ml, exhibiting comparable activity to diclofenac.

The sourdough extract achieved a maximum inhibition of 22.93 ± 0.73% at 1600 µg/ml, exceeding the maximum inhibition of 14.78 ± 2.95% observed with diclofenac (*p* < 0.001). This indicates that the sourdough extract possesses a greater ability to stabilize red blood cell membranes. Furthermore, the inhibitory activity of the sourdough extract increased significantly with increasing concentrations, suggesting a concentration-dependent effect.

Similar to *Citrus bergamia* essential oil’s membrane-disrupting action against bacteria [22], the sourdough extract demonstrated membrane-stabilizing effects on red blood cells, exceeding diclofenac’s protective capacity against hemolysis. The stabilization of red blood cell membrane assay serves as an in vitro model for assessing anti-inflammatory activity, as red blood cell membranes are analogous to the lysosomal membranes of inflammatory cells. Lysosomal stabilization is crucial for limiting inflammation by preventing the release of lysosomal constituents, such as bactericidal enzymes and proteases, from activated neutrophils during microbial infections [23]. These enzymes contribute to various cellular disorders, and their extracellular activity can contribute to both acute and chronic inflammation. The sourdough extract exhibited significant red blood cell membrane stabilization, surpassing the effect of diclofenac. This stabilization was concentration-dependent, with a maximum of 22.93 ± 0.73% achieved at a concentration of 1600 µg/ml, compared to 14.78 ± 2.95% for diclofenac at the same concentration. This suggests that the sourdough extract’s ability to stabilize red blood cell membranes may contribute to its overall anti-inflammatory properties.

### Inhibition of Protease Activity

To further investigate the anti-inflammatory potential of the sourdough extract, we assessed its effect on protease activity using a trypsin-based assay. Fig. S2C illustrates the inhibitory effect of the sourdough extract on protease activity, demonstrating a slightly higher level of inhibition compared to diclofenac at various concentrations (100 to 1600 µg/ml). This inhibitory action increases significantly with higher concentrations, indicating a concentration-dependent effect. At a concentration of 1600 µg/ml, the sourdough extract achieved an inhibition of 26.70 ± 1.36%, surpassing the maximum inhibition of 24.64 ± 0.17% observed with diclofenac (*p* < 0.05).

This protease inhibition activity supports the extract’s potential anti-inflammatory properties, as trypsin, a key mediator of inflammation, is a serine protease found abundantly in neutrophil lysosomes. Leukocyte proteases are known to contribute to tissue damage during inflammatory reactions, and protease inhibitors, such as flavonoids, have demonstrated protective effects. While flavonoids have been shown to inhibit key inflammatory mediators, including prostaglandins, nitric oxide, and C-reactive protein, data on their anti-protease activity remain limited [24, 25].

### Antioxidant Activity

#### Hydrogen Peroxide (H_2_O₂) Reducing Activity

Fig. S3A illustrates the hydrogen peroxide neutralization activity of the sourdough extract across a range of concentrations. The results indicate a concentration-dependent increase in the extract’s ability to reduce H₂O₂.

At a concentration of 1600 µg/mL, the sourdough extract exhibited a significant (81.61 ± 2.98%) reducing capacity for H₂O₂, considerably higher than the standard gallic acid (12.27 ± 0.10%) at the same concentration (*p* < 0.0001). This difference suggests that the polyphenols responsible for DPPH and H₂O₂ scavenging may differ in their effectiveness against specific radicals [26]. The presence of phenolic compounds capable of donating electrons to H₂O₂ explains the extract’s ability to neutralize H₂O₂.

#### Ferric-reducing Antioxidant Power (FRAP)

The ferric-reducing antioxidant power (FRAP) of the sourdough extract was evaluated using the iron (Fe) reduction assay, with gallic acid serving as a positive control. The results, depicted in Fig. S3B, demonstrate a unique pattern of antioxidant activity.

While the FRAP values of the sourdough extract remained relatively stable across the tested concentration range (100–1600 µg/mL), exhibiting a slight decrease from 96.26 ± 0.03 to 95.63 ± 0.06%, this consistent high level of activity was significantly greater than that observed for gallic acid, which displayed a maximum FRAP value of only 4.43 ± 0.017% at the highest concentration tested (1600 µg/mL). Statistical analysis revealed a highly significant (*p* < 0.0001) concentration-dependent reduction of ferric to ferrous iron by the sourdough extract, indicating a notable ability to donate electrons and act as a reducing agent. The stability in inhibition percentage across increasing concentrations suggests a potential for activity even at lower doses. This could be attributed to the saturation of active sites on the molecules responsible for iron reduction at higher concentrations, leading to competition for binding [9].

The sourdough extract’s observed antioxidant effects are consistent with research highlighting the antioxidant potential of plant-derived compounds. Foss et al. [27] demonstrated the significant antioxidant activity of various herbs and spices, attributing it to their rich content of phenolic acids, flavonoids, and stilbenes. These bioactive compounds effectively neutralize free radicals, mitigating oxidative stress and promoting overall health [27]. Their mechanism of action involves donating hydrogen atoms or electrons, and chelating transition metal ions like iron and copper, thus preventing further radical formation [28]. This aligns with studies on other natural products, such as herbal teas, which, like the sourdough extract, are rich in phenolic compounds. Büyükbalci et al. (2008) showed the high antioxidant capacities of herbal teas, especially green tea, due to their abundant phenolic acid and flavonoid content [29]. Furthermore, the addition of natural flavoring agents like clove or cinnamon, known to enhance antioxidant properties through similar mechanisms of free radical neutralization and hydrogen/electron donation, reinforces the crucial role of phenolic compounds in the sourdough extract’s antioxidant activity.

#### DPPH Radical Scavenging Activity

The antioxidant capacity of the sourdough extract was assessed using the DPPH radical scavenging assay, with ascorbic acid serving as a positive control. A range of concentrations (100–1600 µg/ml) of both the sourdough extract and ascorbic acid were tested, and the percentage reduction of the DPPH radical was measured. As shown in Fig. S3C, the inhibition percentage increased with increasing concentrations of both the extract and the standard. The sourdough extract exhibited a maximum antioxidant effect of 14.29 ± 0.04% at a concentration of 1600 µg/ml. In comparison, ascorbic acid displayed a significantly higher maximum effect of 87.93 ± 0.09% at the same concentration, indicating superior radical scavenging activity (*p* < 0.0001).

Ascorbic acid is well-known for its strong antioxidant and anti-inflammatory properties. It works synergistically with external compounds such as natural polyphenols to enhance free radical neutralization and stimulate cellular antioxidant defenses. This cooperation also contributes to the activation of the Nrf2 factor, thereby reducing inflammation and improving protection against UV-induced skin damage [30].

### Molecular Docking Results

#### Predicted Interactions of Sourdough Components with the IL-1β Signaling Complex

The molecular docking study focused on evaluating the binding interactions of selected compounds against the IL-1β signaling complex (PDB ID: 4DEP). The results are summarized in Table S3, which provides the predicted interactions, binding energies, and distances between the ligands and specific amino acids in the protein. Thymol exhibited the most favorable binding energy of -6.6 kcal/mol. Key interactions included conventional hydrogen bonds with MET128 (1.92 Å) and CYS125 (1.69 Å), as well as π-anion interactions with GLU129 (4.46 Å) and ASP162 (3.87 Å). Alkyl interactions were observed with VAL124 (5.43 Å), LYS132 (4.32 Å), and LEU138 (4.43 Å), along with a π-alkyl interaction involving HIS30 (4.82 Å). These interactions suggest a strong and stable binding conformation of thymol within the active site of the IL-1β signaling complex (Fig. S4).

Phthalic acid showed a binding energy of -5.5 kcal/mol, interacting primarily through conventional hydrogen bonds with LYS16 (2.46 Å, 2.47 Å), MET128 (1.60 Å), and CYS125 (3.03 Å), as well as a carbon hydrogen bond with HIS30 (2.19 Å). Additional π-anion interactions were observed with ASP162 (3.99 Å), contributing to the stability of the ligand-protein complex (Fig. S5).

Lactic acid and glycerin demonstrated lower binding energies of -4.5 and − 3.9 kcal/mol, respectively. Both compounds formed multiple conventional hydrogen bonds, with MET128 being a common interaction site for both ligands. Lactic acid also interacted with GLU129 and GLN126, while glycerin established additional carbon hydrogen bonds with GLU128, CYS125, and TYR127 (Fig. S6 and S7).

The anti-inflammatory activities of these compounds, identified through GC-MS analysis, have garnered particular attention. The high inhibition activities observed in in vitro assays correlate with the biological activities of these compounds. A molecular docking analysis revealed that thymol and phthalic acid exhibited strong interactions with the IL-1β signaling pathway. These interactions suggest potential anti-inflammatory effects by targeting IL-1β, a key regulator in inflammatory processes.

Interleukin-1 (IL-1) family cytokines play a crucial role in both innate and adaptive immunity. They exert pro-inflammatory effects by first binding to a primary receptor and subsequently interacting with a receptor accessory protein, forming a heterotrimeric complex that facilitates signal transduction [31].

The molecular interactions of bioactive compounds present in sourdough extract with inflammatory pathways were evaluated at the molecular level. Molecular docking analyses revealed that thymol (-6.6 kcal/mol) and phthalic acid (-5.5 kcal/mol) exhibited high binding affinities toward the IL-1β signaling complex, suggesting that these compounds may play a potential role in inflammatory processes. However, it should be noted that molecular docking analyses provide only theoretical predictions, and these results need to be validated in biological systems. The observed anti-inflammatory effects of the extract may not solely be attributed to thymol and phthalic acid; other components, such as lactic acid and glycerin, could also potentially contribute to the modulation of inflammatory responses. Specifically, lactic acid (-4.5 kcal/mol) and glycerin (-3.9 kcal/mol) were shown to exhibit moderate binding affinities toward the IL-1β signaling complex. These compounds have lower binding energies compared to thymol and phthalic acid, suggesting that their effects on inflammatory processes may be more limited. These findings indicate that the components present in sourdough extract may modulate inflammatory pathways and could be considered as potential functional food ingredients. However, further in vitro and in vivo studies are needed to investigate the bioavailability, metabolic stability, and physiological effects of these compounds in more detail. Specifically, validating the molecular docking data through cellular and animal model studies is a critical step to determine the therapeutic potential of these compounds. Signals transmitted through IL-1β receptors activate cellular pathways such as NF-κB and MAPK. These pathways modulate the expression of key mediators and genes involved in inflammation [32]. The potential of thymol and phthalic acid to inhibit these pathways could lead to the suppression of inflammatory processes, thereby facilitating the regulation of inflammation. According to the molecular docking results, the studied ligand, agathisflafone, exhibited a binding affinity of − 7.9 kcal/mol towards the IL-1β target biomacromolecule [33]. This value is higher compared to thymol (− 6.6 kcal/mol) and phthalic acid (− 5.5 kcal/mol). Additionally, compounds such as lactic acid (− 4.5 kcal/mol) and glycerin (− 3.9 kcal/mol), although showing lower binding affinities, still interact with IL-1β and may contribute to potential inflammatory modulation. These findings suggest that agathisflafone could modulate the IL-1β signaling pathway more effectively, while the weaker binding affinities of other compounds may result in more limited effects. The lower scores observed for 2’-hydroxy flavanone and its derivatives in the case of IL-1β indicate that these compounds do not bind to the active sites of IL-1β [34]. In our study, thymol, phthalic acid, lactic acid, and glycerin interact with IL-1β, indicating that these compounds bind to the active sites of IL-1β. Additionally, the overexpression of IL-1β leads to immunological complications such as arthritis and autoimmune diseases. Currently, there are several drugs on the market that inhibit IL-1β production or affect the signaling cascade to treat inflammation. These include Dexamethasone, Prednisolone, Colchicine, Minocycline, and Chloroquine. These drugs aim to prevent the direct interaction of IL-1β with its receptor. Such approaches can provide valuable insights for drug design to offer more consistent and effective solutions for the treatment of inflammatory diseases [35]. Lactic acid and glycerin, as alternatives to thymol and phthalic acid, make them potential drug candidates to ameliorate these conditions. Our docking study also supports this conclusion.

### Predicted Interactions of Sourdough Components with Nuclear Receptors

The interactions of the identified metabolites with various nuclear receptors were rigorously assessed using the open-access Endocrine Disruptome web server, with a focus on free binding energies. The metabolites were classified into three categories based on their binding affinities: red, yellow/orange, and green. The red category signifies high binding affinity, yellow/orange denotes moderate affinity, and green indicates low affinity.

The results of this analysis provide a detailed depiction of the interaction profiles of the metabolites with nuclear receptors. These findings offer significant insights into the potential biological effects and underlying mechanisms of the metabolites, as detailed in Table S4.

The colored table represents the docking scores of various metabolites against different nuclear receptors. The values highlighted in yellow for “AR an” indicate that these metabolites have a significant binding potential with AR an. The green color for other receptors emphasizes that these metabolites show a lower binding potential with those receptors. In this context, thymol exhibits a high binding potential with AR an, as evidenced by its yellow-highlighted score. Compared to other receptors, thymol shows lower binding scores, suggesting that thymol tends to interact more significantly with AR an and may reflect potential biological effects associated with this interaction. Phthalic acid also demonstrates a high binding potential with AR an, while its binding potential with other receptors is comparatively lower. This implies that phthalic acid may have a more pronounced effect on AR and limited interactions with other receptors. Lactic acid and glycerin are similarly highlighted in yellow for AR an, indicating a notable binding potential with AR an. However, their binding potentials with other receptors are lower. Specifically, the lower binding energies of lactic acid and glycerin suggest that these metabolites may have limited effects in interactions with other nuclear receptors. These findings indicate that metabolites with high binding potential to specific nuclear receptors can establish stronger interactions with targeted proteins. This, in turn, may influence potential biological and pharmacological effects.

## Conclusion

This study underscores the significant anti-inflammatory and antioxidant potential of sourdough extract, highlighting its promise as a functional food with health-promoting properties. In vitro assays demonstrated the extract’s ability to effectively inhibit key markers of inflammation, including protein denaturation, protease activity, and red blood cell hemolysis, while also exhibiting robust antioxidant activity in DPPH, H₂O₂, and FRAP assays. These findings suggest that sourdough extract may play a protective role against cellular damage and oxidative stress, which are central to the pathogenesis of various chronic diseases.

Molecular docking studies provided mechanistic insights, revealing that bioactive compounds such as thymol and phthalic acid exhibit strong binding affinities with the IL-1β signaling complex, a key regulator of inflammatory pathways. This interaction suggests that these compounds may modulate inflammatory responses, further supporting the extract’s therapeutic potential.

By elucidating the molecular mechanisms underlying sourdough’s health benefits and identifying its key bioactive components, this study lays the groundwork for future investigations into its use as a natural, dietary intervention for managing inflammation and oxidative stress-related conditions. These efforts could ultimately contribute to the development of novel functional foods and nutraceuticals aimed at promoting human health and preventing disease.

Future research should focus on validating these findings through in vivo models to assess the extract’s efficacy in reducing inflammation and oxidative stress in living systems. Additionally, exploring the synergistic effects of other phytochemicals present in sourdough extract could provide a more comprehensive understanding of its bioactivity. Investigating the extract’s potential in disease models, such as cancer, cardiovascular diseases, and metabolic disorders, would also be valuable. Furthermore, optimizing dosage, formulation, and delivery methods will be critical for translating these findings into practical therapeutic applications.

## Electronic Supplementary Material

Below is the link to the electronic supplementary material.

Supplementary Material 1

## Data Availability

No datasets were generated or analysed during the current study.
