# Supplementary material for: Unlocking the Anti-Inflammatory Potential of Sourdough: Phytochemical Profile, Functional Investigation, and Molecular Docking Insights into Key Bioactive Compounds
Source: Plant Foods Hum Nutr. 2025 Apr 8;80(2):108. doi: 10.1007/s11130-025-01345-4 (PMC11978714; doi:10.1007/s11130-025-01345-4)
Supplement: Supplementary file 1 — Supplementary Material 1 [file 11130_2025_1345_MOESM1_ESM.docx]

**Materials and Methods**

**Sourdough Preparation**

The sourdough was prepared by adding spring water to whole durum wheat (Triticum durum) semolina of Algerian origin (1/1 weight/volume) to get a homogeneous mixture. The recipient containing the mixture was covered with a clean towel and kept at room temperature for 24 hours. The dough is then refreshed every 12 hours. During this period, the development of bubbles and a double volume were observed. The pH measurement was conducted using a pH meter (HI2210 pH Meter, HANNA Instruments). To obtain a dried sourdough powder, a thin layer of sourdough was spread onto baking paper and dried in an incubator (Memmert) at 35°C, shielded from light, until completely dry. The dried sourdough was then ground into a fine powder using an electric grinder (Type CB-MAC-7020). The resulting powder was stored in a sterile glass bottle, covered with aluminum foil, and kept at 5°C until use [1].

*2.2. Extract preparation*

Extraction is the main step in the purification of phenolic compounds before analysis and application, as it allows the concentration of these compounds and reduces the interfering components. One of the non-conventional extraction techniques is the application of ultrasound, which intends to boost extraction efficiency and yields of phenolic compounds from cereals.

The sourdough extract was freshly prepared before each test following the method of [2] with minor modifications. A specific mass of sourdough powder was added to a measured volume of distilled water to achieve the desired concentration. The mixture was then vortexed (Shaker Heidolph REAX top) for 5 minutes, sonicated (BANDELIN SONOREX) for three cycles (10 seconds on/ 10 seconds off), and gently shaken for 1 hour. The samples were centrifuged (Hettich EBA 20 Centrifuge) for 10 minutes at 5400 rpm. The supernatant was then collected and filtered with filter paper (Whatman No. 3). The resulting filtrate was used for immediate analysis.

**Phytochemical Screening**

**Determination of Total Phenolic Compounds**

The total phenolic content of the extract was determined using the Folin-Ciocalteu method adapted from [3]. Briefly, 0.2 ml of extract (250 μg/ml) or standard solution was mixed with 1.5 ml of diluted Folin-Ciocalteu reagent (1/10). After a 5-minute incubation at room temperature in the dark, 1.5 ml of a 7.5% sodium carbonate (Na₂CO₃) solution was added. The mixture was then incubated for 90 minutes at room temperature in the dark. Absorbance was measured at 750 nm against a blank using a SPECORD® 50 PLUS spectrophotometer. A standard curve was constructed using various concentrations of gallic acid under identical conditions. The total phenolic content was expressed as milligrams of gallic acid equivalents per gram of crude extract (mg GAE/g). Each analysis was performed in quadruplicate, and the average value was reported.

**Determination of Total Flavonoids**

The total flavonoid content was determined spectrophotometrically using a modified method adapted from [4]. An aliquot of 1.5 ml of the 250 µg/ml extract or standard solution was mixed with 1.5 ml of 2% aluminum chloride (AlCl₃) solution. After a 30-minute incubation at room temperature, the absorbance was determined at 430 nm against blank using the SPECORD® 50 PLUS spectrophotometer. A standard curve was constructed using various concentrations of quercetin under identical conditions. The total flavonoid content was expressed as milligrams of quercetin equivalents per gram of crude extract (mg QE/g). Each analysis was performed in quadruplicate, and the average value was reported.

**Determination of Flavonols**

The flavonol content was determined using a modified colorimetric method described by [5]. An aliquot of 1 ml of 250 µg/ml extract was mixed with 1 ml of 2% aluminum chloride (AlCl_3_), followed by the addition of 3 ml of a 5% sodium acetate solution. The mixture was incubated for 30 min at room temperature in the dark. Absorbance was measured at 440 nm using the SPECORD® 50 PLUS spectrophotometer. A standard curve was constructed using various concentrations of quercetin under identical conditions. The total flavonoid content was expressed as milligrams of quercetin equivalents per gram of crude extract (mg QE/g). Each analysis was performed in quadruplicate, and the average value was reported.

**GC-MS Analysis**

The phytochemical analysis of the methanolic extract was performed using a Shimadzu QP2010 mass spectrometer equipped with electron impact ionization (70 eV). A 1 µL sample was injected onto an SE capillary column (0.25 µm × 25 mm) coupled to a quadrupole mass analyzer. The oven temperature was programmed to increase from 90°C to 240°C at a rate of 3°C/min, with a 2-minute hold time at the final temperature, resulting in a total run time of 52 minutes. The source and interface temperatures were maintained at 200°C and 250°C, respectively. Helium was used as the carrier gas with a flow rate of 1.2 mL/min and a split ratio of 20:1. The scan time and mass range were set to 0.50 s and 40-350 m/z, respectively. Compound identification was achieved by comparing the obtained mass spectra with the NIST mass spectral library using the GC/MS data system. Percentage composition was determined based on peak area.

**In Vitro Evaluation of Sourdough Extract’s Anti-Inflammatory Activity**

**Protein Denaturation Test**

Inflammation is a complex process that involves increased protein denaturation. The denaturation of albumin can lead to the formation of antigens, triggering a type III hypersensitivity reaction and contributing to inflammation. Therefore, inhibiting protein denaturation has been established as an *in vitro* assay for evaluating anti-inflammatory activity[6].

The anti-inflammatory potential of the sourdough extract was evaluated using an ovalbumin denaturation inhibition assay adapted from [7]. A reaction mixture (5 ml) containing 0.2 mL of fresh chicken egg albumin, 2.8 mL of phosphate buffer (pH 6.4), and 2 ml of various concentrations (100 to 1600 µg/ml) of the sourdough extract or standard drug (diclofenac 50 mg/ml) was incubated for 15 minutes at room temperature. A control, containing 2 ml of distilled water instead of the extract, was prepared concurrently. The reaction mixtures were then heated at 70°C for 5 minutes, followed by cooling to room temperature. Absorbance was measured at 660 nm using a spectrophotometer (SPECORD® 50 PLUS) against a blank prepared under the same conditions. The control represented 100% ovalbumin denaturation. The percentage of ovalbumin denaturation inhibition was calculated using the following equation:

Inhibition (%) = ​​×100 (Abs control​ - Abs sample)/Abs control​

Abs control is the absorbance of the control.

Abs sample is the absorbance of the sourdough extract sample.

**Cell membrane stabilization test**

The principle of this method relies on the ability of the extract to stabilize red blood cell membranes against heat-induced hemolysis, compared to a standard solution. Red blood cell membrane stabilization was evaluated using a modified procedure based on [8]. Blood was collected from a healthy human volunteer and mixed with an equal volume of sterile Alsever's solution (2% glucose, 0.8% sodium citrate, 0.5% citric acid, and 0.42% sodium chloride in distilled water). The mixture was centrifuged at 3000 rpm for 10 minutes, and the pellet was washed three times with physiological saline (0.9%). The volume of packed red blood cells was measured and reconstituted as a 10% v/v suspension in physiological saline. This suspension was stored at 4°C until use. The hemolysis assay involved preparing mixtures containing 1 mL of phosphate buffer (pH 7.4, 0.15 M), 2 mL of hypotonic saline (0.42%), 0.5 mL of red blood cell suspension (10%), and 0.5 mL of either the extract or the standard drug diclofenac at various concentrations (100 to 1600 μg/mL). A control group (distilled water instead of extract or drug) was also included to induce 100% hemolysis. These mixtures were incubated at 56°C for 30 minutes, and then centrifuged at 3000 rpm for 10 minutes. The supernatants were collected, and their absorbance was measured at 560 nm using a spectrophotometer (SPECORD® 50 PLUS). The percentage of red blood cell membrane stabilization (inhibition of hemolysis) was calculated using the following formula:

The percentage of stabilization (%) = (Abs control - Abs sample/Abs control) × 100

Abs control is the absorbance of the control.

Abs sample is the absorbance of the sourdough extract sample.

**Protease Inhibitory Test**

To assess the inhibitory activity of the extract against trypsin, a modified version of the methods described by [9] was adopted. The reaction mixture (2 mL) contained 0.06 mg of trypsin and 1 mL of 20 mM Tris-HCl buffer (pH 7.4). Diclofenac was used as the standard, and distilled water served as the negative control. After incubating the reaction mixture at 37°C for 5 minutes, 1 mL of 0.8% (w/v) casein prepared in buffer solution was added. The mixture was further incubated for an additional 20 minutes, followed by the addition of 2 mL of 70% acetic acid to stop the reaction. The suspension was centrifuged at 3000 rpm for 10 minutes, and the absorbance of the supernatant was measured using a spectrophotometer (SPECROD® 50 PLUS) at 210 nm against a buffer blank. Each experiment was conducted in triplicate. The percentage of inhibition of protease activity was calculated using the following formula:

Inhibition (%) = ​​×100 (Abs control - Abs sample)/Abs control

Abs control is the absorbance of the control.

Abs sample is the absorbance of the sourdough extract sample.

**In Vitro Evaluation of Sourdough Extract’s Antioxidant Activity**

**DPPH Free Radical Scavenging Assay**

For our investigation, the [10] methodology was used. Therefore, 1 mL of an ethanolic DPPH solution (100 µM) was mixed with 500 µL of sourdough extract at various doses (100 to 1600 µg/mL). Using a spectrophotometer (SPECROD® 50 PLUS), absorbance was measured at 517 nm following agitation and 30 minutes of room temperature, dark incubation. The negative control was a solution that contained 1 mL of DPPH and 500 µL of ethanol. The same quantities of ascorbic acid were utilized as a standard as they were for the sourdough extract. Every assay was run in triplicate. The antiradical activity is expressed as the percentage reduction of DPPH• using the following formula:

Percentage reduction of DPPH (%) = ​​×100 (Abs control - Abs sample)/Abs control

Abs control is the absorbance of the control.

Abs sample is the absorbance of the sourdough extract sample.

**Hydrogen Peroxide Reducing Assay**

The capacity of the sourdough extract to neutralize hydrogen peroxide was determined using the [11]method, with some modifications. To prepare the test tubes, 2.0 mL of sourdough extract (100 to 1600 μg/mL) was mixed with 1.2 mL of 40 mM hydrogen peroxide in phosphate buffer solution (pH 7.4; 0.1 M). A blank was created in the same way, but without hydrogen peroxide. A control was prepared simply using hydrogen peroxide (no extract). After 10 minutes of incubation, absorbance at 230 nm was measured using a spectrophotometer (SPECROD® 50 PLUS). The percentage of hydrogen peroxide reduction is calculated using the following formula:

Percentage reduction of H_2_O_2_ (%) = ​​×100 (Abs control - Abs sample)/Abs control

Abs control is the absorbance of the control.

Abs sample is the absorbance of the sourdough extract sample.

**Ferric-Reducing Antioxidant Power (FRAP) Assay**

The reducing power of the extracts was measured using the procedure described by [12]**.** To each sample, 0.5 mL of extract at different concentrations (100 to 1600 μg/mL) was mixed with 1.25 mL of 0.2 M phosphate buffer (pH 6.6) and 1.25 mL of 1% potassium ferricyanide K3[Fe(CN)6]. The mixture was incubated for 20 minutes at 50°C in the dark. Subsequently, 1.25 mL of trichloroacetic acid (10%) was added and centrifuged at 3000 rpm for 10 minutes. 1.25 mL of the supernatant was extracted, diluted with 1.25 mL of distilled water, and 0.25 mL of 0.1% FeCl3 was added. The mixture was incubated at 28°C for 30 minutes to allow color development. Absorbance was measured at 700 nm using a spectrophotometer (SPECROD® 50 PLUS). A positive control containing a standard antioxidant, such as ascorbic acid or gallic acid, was run concurrently under identical conditions to establish a reference point for antioxidant activity. The percentage of Fe reduction is calculated using the following formula:

Reduction (%) = ​​×100 (Abs control - Abs sample)/Abs control

Where:

Abs control is the absorbance of the control.

Abs sample is the absorbance of the sourdough extract sample.

**Molecular Docking Studies**

**Exploration of Protein-Ligand Interactions**

Molecular docking, an important computational tool in drug design, employs algorithms to predict the binding affinities and interactions of ligands such as thymol, phthalic acid, lactic acid, and glycerin with target proteins. The application of this method contributes to the identification of potential drug candidates, optimization of molecular structures, and understanding of complex molecular recognition mechanisms that are critical for effective pharmaceutical design.

The protein (PDB ID: 4DEP) was downloaded from the Protein Data Bank (PDB). It was prepared by removing water molecules and heteroatoms and adding polar hydrogens using BIOVIA Discovery Studio Visualizer 2021. After selecting the embedded ligand, a grid was generated through the “Define and Edit Binding Site – From Current Selection” tool in BIOVIA Discovery Studio Visualizer 2021[13]. The protein was saved in .pdb format and converted to. pdbqt format using AutoDock Tools (v-1.5.7). For the internal ligand, it was extracted from the grid box and pasted into a new window, then saved in .pdb format. It was also converted to. pdbqt format using AutoDock Tools (v-1.5.7). The drawn ligand underwent energy minimization, was saved in .pdb format, and subsequently converted to. pdbqt format using AutoDock Tools (v-1.5.7). Docking scores were obtained for specific poses and were utilized for scoring analysis from the initial pose. Log and output files were generated. To analyze amino acid interactions, BIOVIA Discovery Studio Visualizer 2021 was employed [14].

**Assessment of Metabolite Interactions with Nuclear Receptors**

The metabolic activity of 15 nuclear receptors was predicted using the Endocrine Disruptome web server **(http://endocrinedisruptome.ki.si/, accessed on 15 September 2024)**. This server simulates the docking of each metabolite with crystal structures of various nuclear receptors, including androgen receptors (AR), oestrogen receptors α and β (ER α/β), glucocorticoid receptor (GR), liver X receptors α and β (LXR α/β), mineralocorticoid receptor (MR), peroxisome proliferator-activated receptors α, β, and γ (PPAR α, PPAR β, and PPAR γ), progesterone receptor (PR), retinoid X receptor α (RXR α), and thyroid receptors α and β (TR α and TR β). The results from the web server are categorized into three levels: red indicates a high binding potential, orange and yellow suggest a moderate binding probability, and green signifies a low likelihood of binding to the receptors**.**

**Statistical Analysis**

Data are presented as mean ± standard deviation. Statistical analysis was performed using GraphPad Prism version 5.00 for Windows (GraphPad Software, San Diego, CA, USA). Differences between groups were assessed using one-way analysis of variance (ANOVA) followed by Bonferroni post-hoc multiple comparisons. Significance levels were set at **p* < 0.05, ***p* < 0.01, ****p* < 0.001, and *p* > 0.05 for non-significant differences.

**References**

1. Nouska C, Hatzikamari M, Matsakidou A, et al (2023) Enhancement of Textural and Sensory Characteristics of Wheat Bread Using a Chickpea Sourdough Fermented with a Selected Autochthonous Microorganism. Foods 12:3112. https://doi.org/10.3390/FOODS12163112/S1

2. Gabriele M, Cavallero A, Tomassi E, et al (2024) Assessment of Sourdough Fermentation Impact on the Antioxidant and Anti-Inflammatory Potential of Pearl Millet from Burkina Faso. Foods 2024, Vol 13, Page 704 13:704. https://doi.org/10.3390/FOODS13050704

3. Othman A, Ismail A, Abdul Ghani N, Adenan I (2007) Antioxidant capacity and phenolic content of cocoa beans. Food Chem 100:1523–1530. https://doi.org/10.1016/J.FOODCHEM.2005.12.021

4. Djeridane A, Yousfi M, Nadjemi B, et al (2006) Antioxidant activity of some algerian medicinal plants extracts containing phenolic compounds. Food Chem 97:654–660. https://doi.org/10.1016/J.FOODCHEM.2005.04.028

5. Shehata F, Fathy A, Abdelhameed M, Moustafa SF (2009) Preparation and properties of Al2O3 nanoparticle reinforced copper matrix composites by in situ processing. Mater Des 30:2756–2762. https://doi.org/10.1016/J.MATDES.2008.10.005

6. Mirke NB, Shelke PS, Malavdkar PR, Jagtap, PN (2020) In vitro protein denaturation inhibition assay of Eucalyptus globulus and Glycine max for potential anti-inflammatory activity. Innovative Pharmacy and Pharmacotherapy 8: 28-31. https://doi.org/10.31690/ipp.2020.v08i02.003

7. Dharmadeva S, Galgamuwa L, Prasadinie C, Kumarasinghe N (2018) In vitro anti-inflammatory activity of Ficus racemosa L. bark using albumin denaturation method. AYU (An international quarterly journal of research in Ayurveda) 39:239. https://doi.org/10.4103/AYU.AYU_27_18

8. Kosala K, Widodo MA, Santoso S, Karyono S (2018) In vitro and In vivo Anti- inflammatory Activities of Coptosapelta flavescens Korth Root’s Methanol Extract. Journal of Applied Pharmaceutical Science 8(09): 042-048.

9. Sakat S, Juvekar AR, Gambhire MN (2010) In vitro antioxidant and anti-inflammatory activity of methanol extract of Oxalis corniculata Linn. J. Pharm. Pharm. Sci 2(1): 46-155.

10. Ancerewicz J, Migliavacca E, Carrupt PA, et al (1998) Structure–Property Relationships of Trimetazidine Derivatives and Model Compounds as Potential Antioxidants. Free Radic Biol Med 25:113–120. https://doi.org/10.1016/S0891-5849(98)00072-0

11. Brand-Williams W, Cuvelier ME, Berset C (1995) Use of a free radical method to evaluate antioxidant activity. LWT - Food Science and Technology 28:25–30. https://doi.org/10.1016/S0023-6438(95)80008-5

12. Chew YL, Goh JK, Lim YY (2009) Assessment of in vitro antioxidant capacity and polyphenolic composition of selected medicinal herbs from Leguminosae family in Peninsular Malaysia. Food Chem 116:13–18. https://doi.org/10.1016/J.FOODCHEM.2009.01.091

13. Biovia (2015) BIOVIA Discovery Studio Visualizer (Version 4.5) Dassault Systèmes, San Diego.

14. Trott O, Olson AJ (2010) AutoDock Vina: Improving the speed and accuracy of docking with a new scoring function, efficient optimization, and multithreading. J Comput Chem 31:455–461. https://doi.org/10.1002/JCC.21334

**Table S1: Total phenolic, flavonoid, and flavonol content (mg/g) of the sourdough extract.** Values represent means ± standard deviations (n = 3 replicates).

|  | **Total phenolics content** | **Total flavonoids content** | **Total flavanols content** |
| --- | --- | --- | --- |
| Sourdough | 0.276 ± 0,0196 | 0.048 ± 0,0006 | 0.013 ± 0,008 |

**Table S2:** Qualitative and quantitative compositions of the sourdough extract.

| **Peak** | **IUPAC name** | **Chemical name** | **Percentage %** | **M/Z** |
| --- | --- | --- | --- | --- |
| **6** | 1,2,3-propanetriol | Glycerin (C_3_H_8_O_3_) | (37.99%) | 61.00 |
| **4** | (S)-2-Hydroxypropanoic acid | Lactic acid (C3H6O3) | (24.27%) | 45.00 |
| **12** | Di-n-octyl Phthalate | Phthalic acid(C_24_H_38_O_4_) | (20.10%) | 149.00 |
| **7** | 5-méthyl-2-(propan-2-yl)-phénol | Thymol(C_10_H_14_O) | (5.65%) | 135.05 |
| **10** | 2H-Indol-2-one,1-(2,6 dichlorophenyl)-1,3-dihydro- | (C_14_H_9_C_l2_NO) | (3.55%) | 214.00 |
| **11** | Cyclopropaneoctanoic acid,2-[[2-[(2-ethycyclopropyl)methyl]cyclopropyl]methyl]-, methyl ester | Docosatrienoic acid (C_22_H_38_O_2_) | (3.07%) | 67.00 |
| **5** | 1,6-Octadien-3-ol,3,7-dimethyl- | Linalol (C_10_H_18_O) | (2.26%) | 71.05 |
| **9** | Ethanol, 2-(9,12-octadecadienyloxy)-, (Z-Z)- | Linoleic acid (C_20_H_38_O_2_) | (1.26%) | 67.00 |
| **8** | Decanoic acid, ethyl ester | Capric acid (C_12_H_24_O_2_) | (0.58%) | 88.00 |
| **2** | 1,3-Propanediol | PDO (C_3_H_8_O_2_) | (0.54%) | 58.05 |
| **3** | Ethyl(dimethyl)ethoxysilane | (C_6_H_16_OSi) | (0.26%) | 103.05 |
| **1** | Decane, 6-ethyl-2-methyl- | (C_13_H_28_) | (0.16%) | 57.05 |

**Table S3.** Predicted interactions of docked conformations of compounds against structure of the IL-1b signaling complex (4DEP)

| Ligand | Protein | Binding Energy  (kcal/mol) | Amino acid | Interacting | Distance |
| --- | --- | --- | --- | --- | --- |
| Thymol | 4DEP | -6.6 | E: MET128:HN -: [001: O1 | Conventional Hydrogen Bond | 1.92 |
|  |  |  | : [001:H9 - E: CYS125:O | Conventional Hydrogen Bond | 1.69 |
|  |  |  | E: GLU129:OE1 -: [001 | Pi-Anion | 4.46 |
|  |  |  | E: ASP162:OD2 -: [001 | Pi-Anion | 3.87 |
|  |  |  | : [001:C7 - E: VAL124 | Alkyl | 5.43 |
|  |  |  | : [001:C10 - E: LYS132 | Alkyl | 4.32 |
|  |  |  | : [001:C10 - E: LEU138 | Alkyl | 4.43 |
|  |  |  | D: HIS30 -: [001:C7 | Pi-Alkyl | 4.82 |
| Phthalic acid | 4DEP | -5.5 | D: LYS16:HZ1 -: [001: O4 | Conventional Hydrogen Bond | 2.46 |
|  |  |  | D: LYS16:HZ2 -: [001: O4 | Conventional Hydrogen Bond | 2.47 |
|  |  | - | E: MET128:HN -: [001: O1 | Conventional Hydrogen Bond | 1.60 |
|  |  |  | E: GLU129:HN -: [001:O1 | Conventional Hydrogen Bond | 3.06 |
|  |  |  | : [001:H3 - D: GLU128:OE1 | Conventional Hydrogen Bond | 2.44 |
|  |  |  | : [001:H4 - E: CYS125:O | Conventional Hydrogen Bond | 3.03 |
|  |  |  | D: HIS30:HE1 -: [001: O4 | Carbon Hydrogen Bond | 2.19 |
|  |  |  | E: ASP162:OD2 -: [001 | Pi-Anion | 3.99 |
| Lactic acid | 4DEP | -4.5 | E: MET128:HN -: [001: O2 | Conventional Hydrogen Bond | 1.65 |
|  |  |  | : [001:H1 - E: GLU129:OE1 | Conventional Hydrogen Bond | 2.18 |
|  |  |  | : [001:H2 - D: GLN126:O | Conventional Hydrogen Bond | 2.14 |
|  |  |  | : [001:C1 - E: VAL124 | Alkyl | 5.44 |
| Glycerin | 4DEP | -3.9 | E: MET128:HN -: [001: O2 | Conventional Hydrogen Bond | 1.73 |
|  |  |  | E: GLU129:HN -: [001: O2 | Conventional Hydrogen Bond | 2.51 |
|  |  |  | : [001:H1 - D: GLN126:O | Conventional Hydrogen Bond | 1.86 |
|  |  |  | : [001:H2 - E: GLU129:OE1 | Conventional Hydrogen Bond | 1.98 |
|  |  |  | : [001:H6 - D: GLN126:O | Conventional Hydrogen Bond | 1.82 |
|  |  |  | E: TYR127: HA -: [001: O2 | Carbon Hydrogen Bond | 2.75 |
|  |  |  | : [001:H3 - D: GLU128:OE1 | Carbon Hydrogen Bond | 2.64 |
|  |  |  | : [001:H4 - E: CYS125:O | Carbon Hydrogen Bond | 2.83 |

**Table S4.** Docking scores of various metabolites against different nuclear receptors

| Metabolites | AR | AR an | ER α | ERβ | GR | LXR α | LXR β | MR | PPAR α | PPAR β | PPAR γ | PR | RXR α | TR α | TR β |
| --- | --- | --- | --- | --- | --- | --- | --- | --- | --- | --- | --- | --- | --- | --- | --- |
| Component |  |  |  |  |  |  |  |  |  |  |  |  |  |  |  |
| **Thymol** | -6.6 | -6.7 | -6.1 | -6.1 | -6.3 | -6.8 | -6.9 | -6.8 | -6.2 | -6.4 | -6.0 | -2.5 | -6.2 | -6.9 | -6.7 |
| **Phthalic acid** | -6.2 | -5.9 | -5.6 | -5.9 | -6.0 | -6.0 | -6.6 | -5.8 | -5.4 | -6.0 | -5.9 | -2.4 | -6.5 | -6.2 | -6.2 |
| **Lactic acid** | -3.9 | -3.8 | -3.6 | -4.0 | -4.1 | -3.4 | -3.6 | -3.5 | -3.7 | -3.5 | -3.5 | -1.8 | -4.4 | -4.1 | -3.6 |
| **Glycerin** | -3.7 | -4.1 | -3.7 | -3.6 | -4.0 | -3.6 | -3.6 | -3.6 | -3.6 | -3.6 | -3.4 | -2.0 | -3.7 | -4.3 | -4.1 |


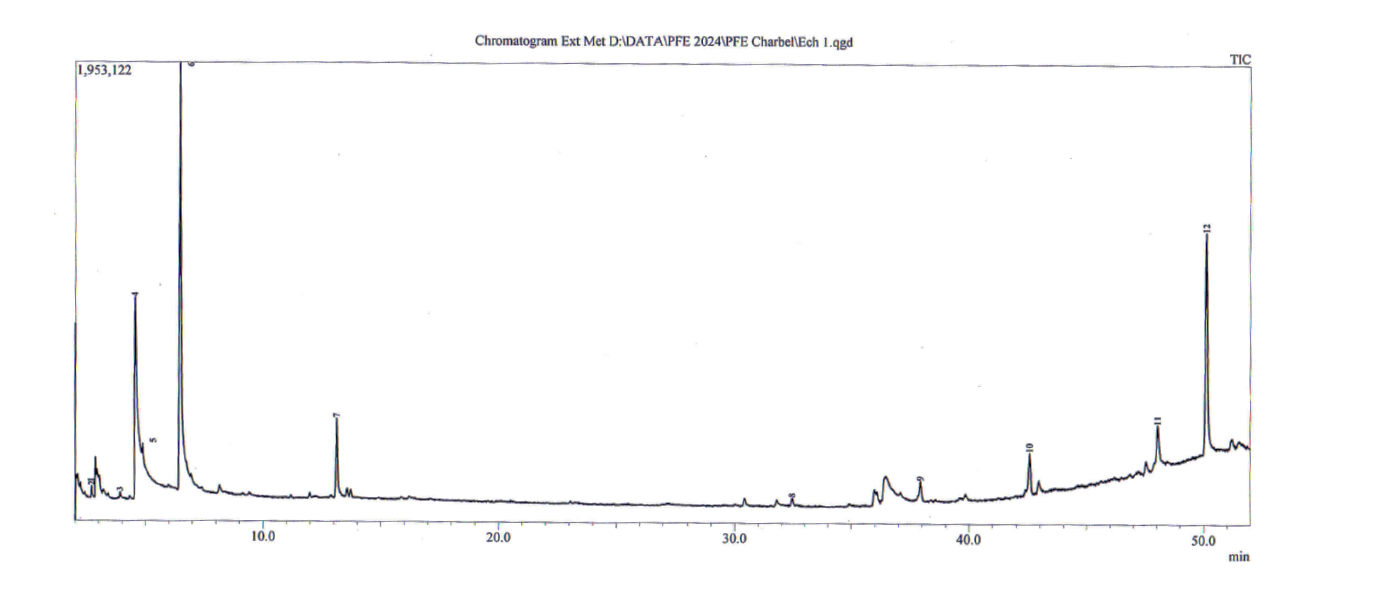


**Fig.S1.** GC/MS profile of sourdough**.** Major components are glycerin (peak 6), lactic acid (peak 4), phthalic acid (peak 12), and thymol (peak 7).


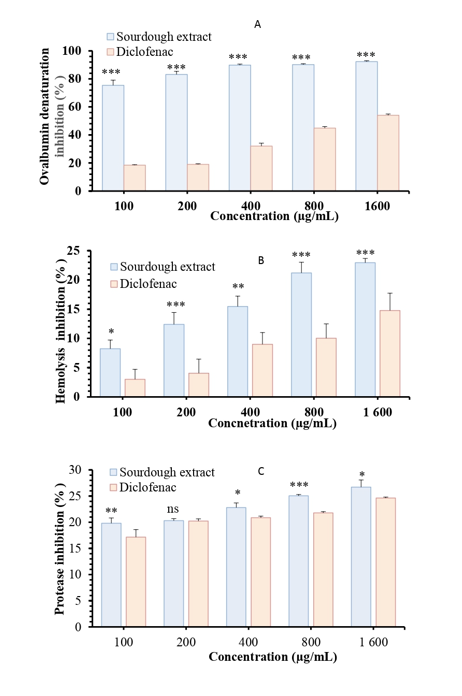


**Fig.S2** In vitro assessment of the anti-inflammatory effects of sourdough extract and diclofenac at different concentrations. A) Ovalbumin denaturation inhibition. B) Hemolysis inhibition. C) Protease inhibition. Results are expressed as means ± standard deviations (n = 3 replicates). Comparison was performed between the sourdough extract and diclofenac at each concentration using analysis of variance (ANOVA) followed by Bonferroni&#39;s multiple comparisons test, *p &lt; 0.05, **p &lt; 0.01, ***p &lt; 0.001, and p &gt; 0.05 for non-significant differences.


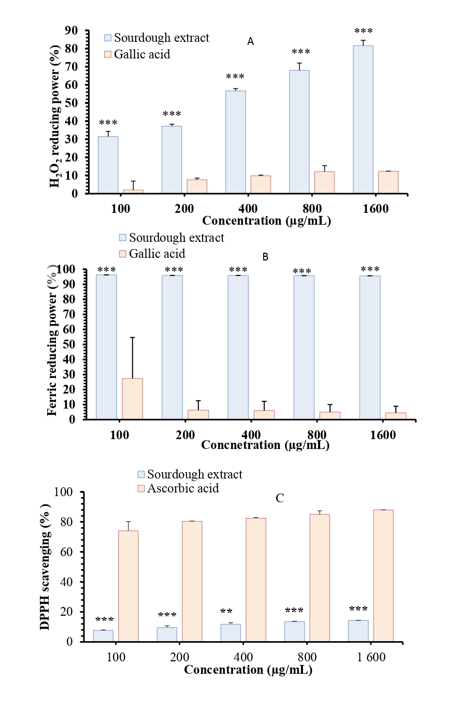


**Fig.S3.** The antioxidant effects of sourdough extract, gallic acid, and ascorbic acid at different concentrations. A) Hydrogen peroxide reducing capacity. B) Ferric reducing power. C) DPPH scavenging activity. Results are expressed as means ± standard deviations (n = 3 replicates). Comparison was performed between the sourdough extract and the standard at each concentration using analysis of variance (ANOVA) followed by Bonferroni's multiple comparisons test, *p < 0.05, **p < 0.01, ***p < 0.001, and p > 0.05 for non-significant differences.


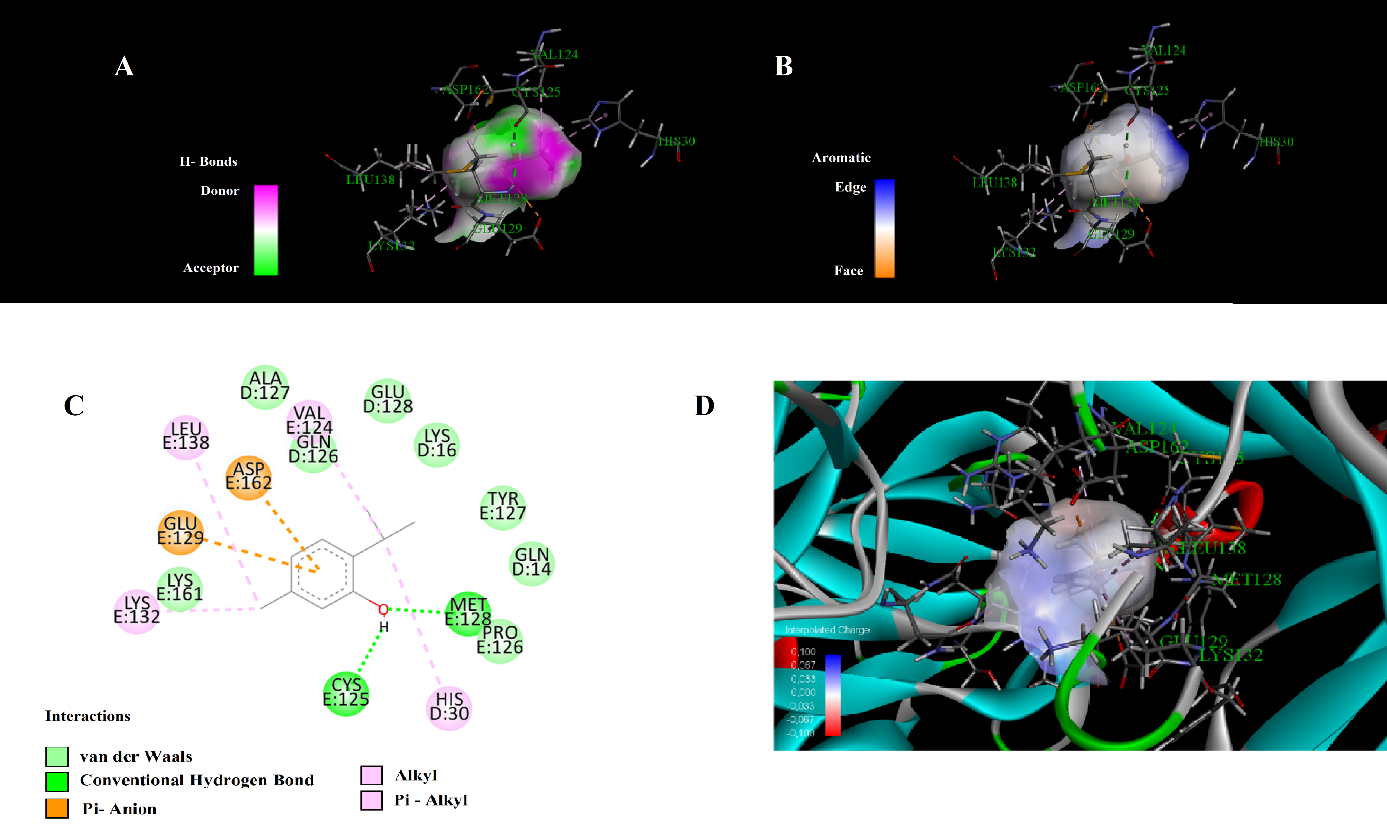


**Fig. S4.** Interactions of thymol with target protein: A) H-bond distribution, B) Aromatic surface representation, C) 2D ligand interaction, D) 3D representation of docked poses.


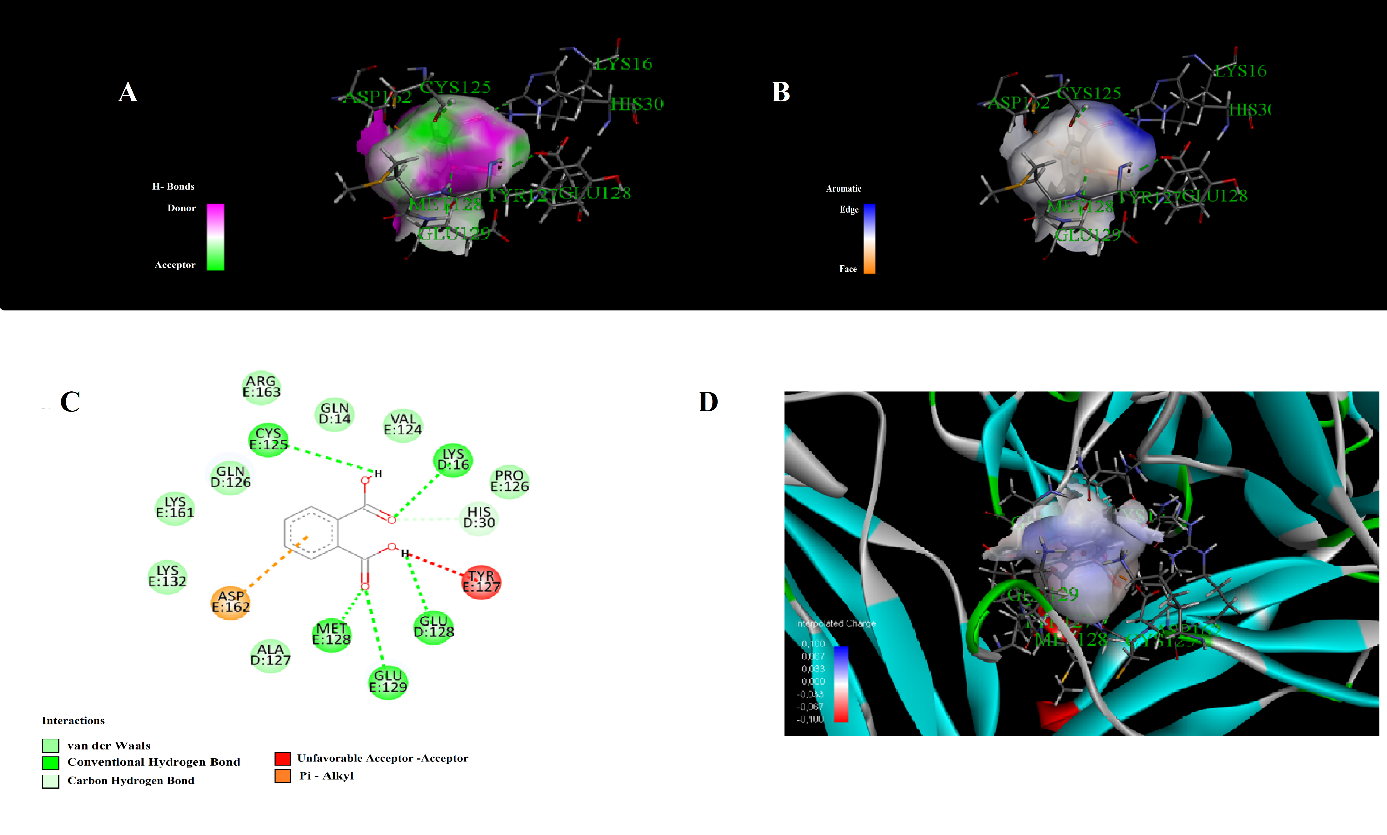


**Fig. S5.** Interactions of phthalic acid with target protein: A) H-bond distribution, B) Aromatic surface representation, C) 2D ligand interaction, D) 3D representation of docked poses


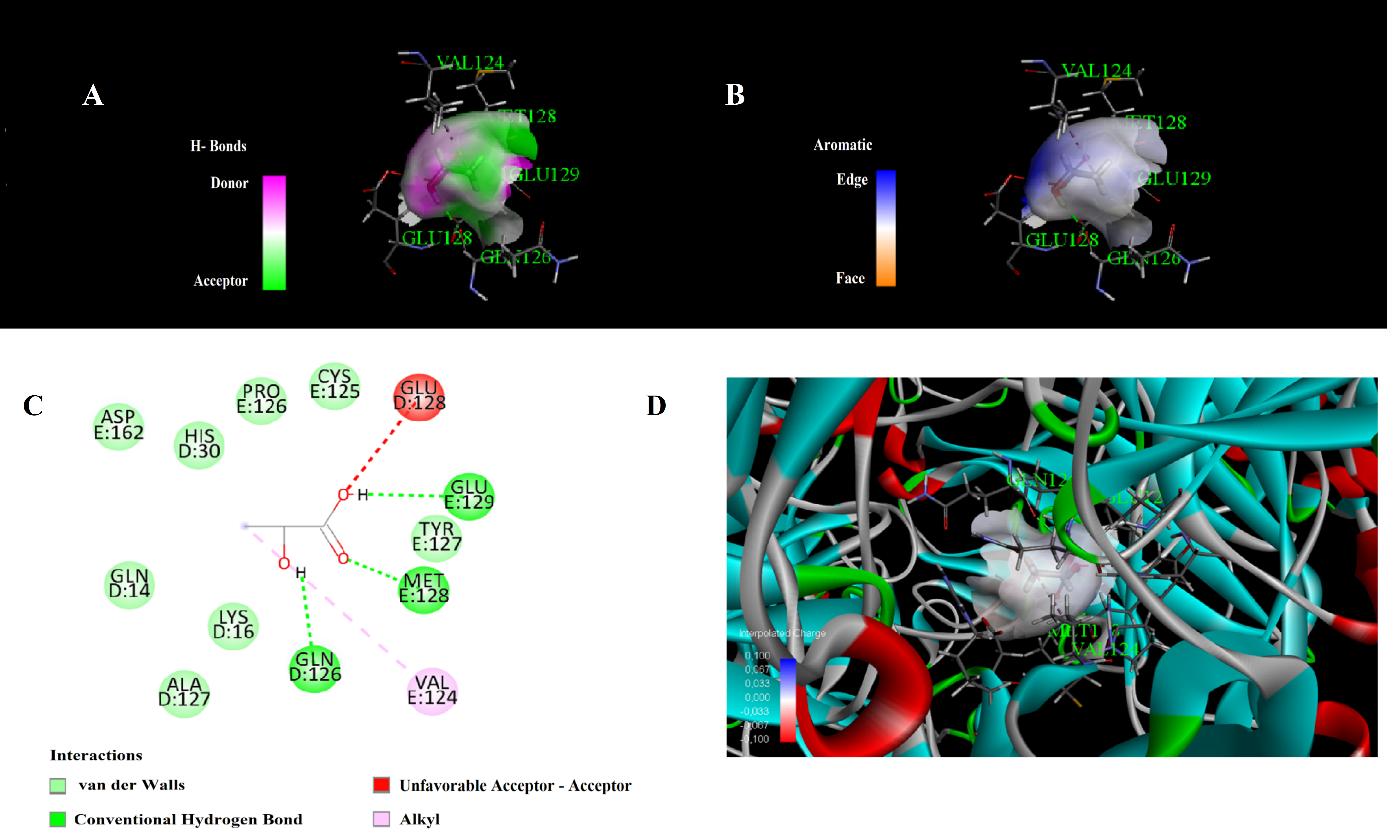


**Fig. S6.** Interactions of lactic acid with target protein: A) H-bond distribution, B) Aromatic surface representation, C) 2D ligand interaction, D) 3D representation of docked poses.


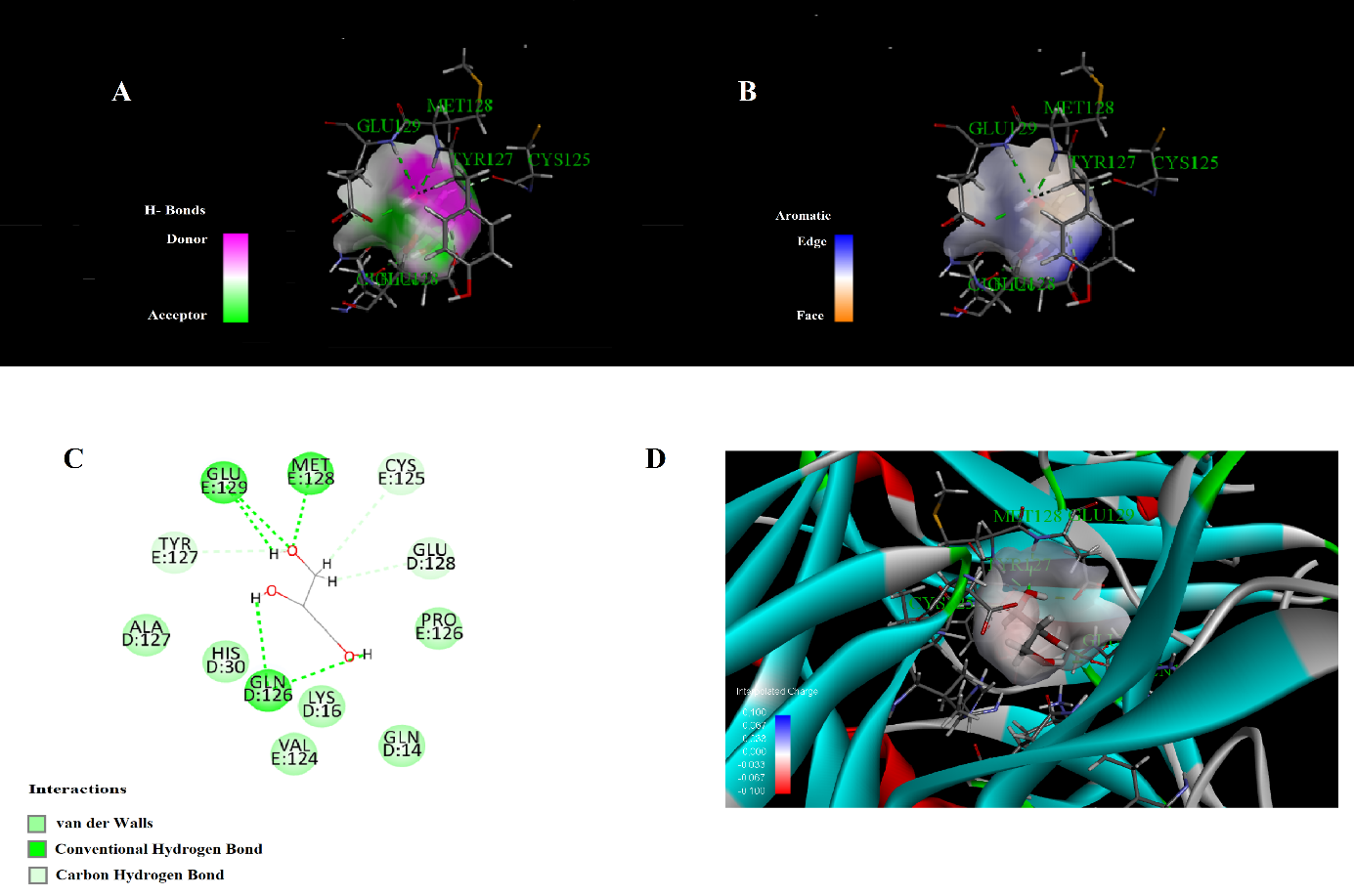


**Fig. S7.** Interactions of glycerin with target protein: A) H-bond distribution, B) Aromatic surface representation, C) 2D ligand interaction, D) 3D representation of docked poses
